# What a wonderful world!

**DOI:** 10.1101/gad.352278.124

**Published:** 2024

**Authors:** Claire Magnon

**Affiliations:** Laboratory of Cancer and Microenvironment-National Institute of Health and Medical Research (INSERM), Institute of Biology François Jacob-Atomic Energy Commission (CEA), University of Paris Cité, University of Paris-Saclay, Paris 75000, France

**Keywords:** brain–body, physiology, symposium

## Abstract

The world of cancer science is moving toward a paradigm shift in making connections with neuroscience. After decades of research on genetic instability and mutations or on the tumor microenvironment, emerging evidence suggests that a malignant tumor is able to hijack and use the brain and its network of peripheral and central neurons as disrupters of homeostasis in the body. Whole-body homeostasis requires brain–body circuits to maintain survival and health via the processes of interoception, immunoception, and nociception. It is now likely that cancer disturbs physiological brain–body communication in making bidirectional brain tumor connections.

## Abstract (precis)

In this Outlook, Magnon discusses how cancer hijacks brain and neuronal networks to influence tumor progression and therapeutic response, using bidirectional interoceptive, immunoceptive and nociceptive communication.

Climate change, migratory movement, increased social conflict, bleak economic climate, major political uncertainty, high risk of terrorist attacks, and pivotal international armed conflict in Eastern Europe were seriously challenging my “*Douce France*,” which was meanwhile preparing and priming Paris for the games of the XXXIII Olympiad, when I was flying to New York to attend the 88th Cold Spring Harbor Laboratory (CSHL) Symposium on Brain Body Physiology. (“*Douce France*” is a song written by Charles Trenet and Leo Chauliac. It was recorded by Charles Trenet in 1947.) While chaos seemed to rule the world, the peace and quiet of CSHL hit me when I just arrived in this secret land, fully dedicated to science and nestled in nature. Later the same day, during the opening evening of the symposium, a violent thunderstorm broke out, as if nature wished us all the best for sharing and building striking biomedical research as several generations of outstanding and committed scientists who came to CSHL previously did. Facing challenges is not only a political task in our restless and chaotic society but also the beating heart of science while the rates of cancer and chronic inflammatory or neurodegenerative disorders are rising across the world.

Understanding how brain–body interplay can maintain homeostasis and health is a fascinating area of research. Visceral organs have the ability to communicate with the brain through afferent sensory or humoral signals in order to inform the central nervous system of any dysfunction of peripheral tissues. In turn, the brain senses and integrates vagal and spinal visceral signals through the interoceptive system to preserve the physiological state of the body. Interestingly, dysregulation of the brain–body connection was shown to affect health, and this has clearly empowered and inspired a new generation of scientists to elucidate mechanisms underlying several diseases. As such, the interoceptive system was shown to be dysfunctional in metabolic or inflammatory diseases. Now, cancer research is making a turning point in setting out on the road toward neuroscience (Magnon and Hondermarck 2023; Amit et al. 2024). The field moves forward, one step further, after decades of research on genetic instability and mutations in cancer cells and later on tumor-associated angiogenesis or immunity. For the first time, the new field of cancer neuroscience that covers how tumors cross-talk with the brain and nerves was recognized as a thematic area in itself in the renowned international symposium at CSHL, focused on neuroscience this year, and this is a striking achievement and reward. This can certainly elicit the interest of cancer scientists to elucidate unanswered questions through the prism of neuroscience, such as how malignant tumors are able to resist or recur despite conventional treatments or antiangiogenic and targeted immune therapies and, if so, to what extent the brain would participate in tumorigenesis and cancer cell dissemination. Would it be too ambitious to envision the brain—and its network of peripheral and central nerves—as the missing pieces of the jigsaw puzzle? And if cancer was finally a neural disorder! This would certainly untangle and possibly solve past, present, or future cancer issues. The literature and the recent discussions at the 88th CSHL Symposium impel us to join efforts in order to take up this challenge (Magnon and Hondermarck 2023).

Significant advances made in the field of cancer neuroscience have determined that peripheral tumors are infiltrated by autonomic and sensory nerve fibers that participate in tumorigenesis and metastasis by interacting with diverse components of the tumor microenvironment (Magnon and Hondermarck 2023). Also, tumors would be able to connect with the central nervous system through afferent sensory neurons, inflammatory cytokines, or neurotrophic factors, suggesting that the brain could sense tumor-induced changes in the body and is likely to process and coordinate tumor signals via different central areas as the brain already does to maintain homeostasis and health (Magnon and Hondermarck 2023). As an example, breast tumors alter the activity of central orexinergic neurons in the lateral hypothalamus, which leads to sleep disruption and metabolic abnormalities, indicating the role of central neuromodulators in metabolic changes induced by peripheral tumors (Borniger et al. 2018). In addition, in a mouse model of breast cancer, corticotropin-releasing hormone neurons in the central medial amygdala connect with the peripheral tumor and affects its growth through the activation of intratumor sympathetic nerves, induced by the lateral paragigantocellular nucleus in the brain (Xiong et al. 2023). Also, we have uncovered that the brain can feed developing breast or prostate adenocarcinomas with doublecortin-expressing neural progenitor cells that are able to egress from the subventricular zone, a neurogenic niche in the brain, and support tumorigenesis. Neural progenitors are released into the bloodstream through the locally permeabilized blood–brain barrier and travel toward the tumor to finally differentiate into adrenergic neurons, suggesting an active brain–tumor communication, or tumor interoception (Mauffrey et al. 2019). Similarly, lung adenocarcinomas are infiltrated by doublecortin-expressing neural cells that display adrenergic properties associated with tumor progression and chemoresistance (Otani et al. 2024). However, further investigation is required to elucidate neural mechanisms at the molecular level underlying the brain–tumor interaction.

Beyond the ability of the central nervous system to control organ functioning, metabolism, or nutritional balance via the interoceptive system, the brain also develops a bidirectional communication with the immune system, called immunoception, that is pivotal to orchestrate peripheral immunity and ensure homeostasis (Chavan et al. 2017; Koren and Rolls 2022). Recently, it was shown that vagal sensory neurons activated by peripheral inflammatory cytokines inform the caudal nucleus of the solitary tract in the brainstem to modulate the innate immune responses (Jin et al. 2024). In addition, a brain–lung circuit regulates allergen-triggered airway hyperreactivity via a vagal activation of Dbh-positive neurons in the nucleus of the solitary tract. Subsequent activation of the nucleus ambiguus finally relays allergen signals to the efferent postganglionic neurons that control airway constriction in the lungs (Su et al. 2024). In cancer, although we are just at the start of uncovering how a bidirectional brain–tumor axis works, it is likely that malignant tumors could entangle the nervous and immune systems in a cross-talk that leads to increased malignancy. Indeed, proinflammatory cytokines are increased in the blood of cancer patients and are significantly associated with cognitive impairment and poor memory. Pharmacological inhibition of inflammation blocks tumor-induced memory loss, suggesting an interaction between the immune and central nervous systems (Magnon and Hondermarck 2023).

In addition, cancer can hijack the visceral nociceptive system that usually allows vagal or spinal sensory neurons to convey pain signaling from peripheral tissues to the brain to maintain homeostasis. It has been shown that sensory nerve fibers innervate peripheral or central malignant tumors and provide support for tumor initiation and progression and also mediate cancer-associated pain (Magnon and Hondermarck 2023). In particular, calcitonin gene-related peptide (CGRP)-expressing sensory neurons in the parabrachial nucleus mediate cancer-induced pain and anorexia but are also involved in modulating immunity in the tumor microenvironment (Magnon and Hondermarck 2023).

The findings in the field of cancer neuroscience are provoking a paradigm shift in our understanding of how cancer develops. Whereas cancer cells seemed only to activate and corrupt noncancer cells to build up the tumor microenvironment, it turns out now that cancer could also attack and damage the entire neural architecture of the body by hijacking the bidirectional communications (i.e., interoception, immunoception, and nociception) that the brain has made with the body to protect or maintain homeostasis and health. Nevertheless, the 88th Symposium at CSHL on Brain Body Physiology has brought hope for making successful advances in cancer research by bringing together lead scientists in brain–body physiology and cancer neuroscience. Although we are at the early stages of research, cancer scientists and neuroscientists have started to meet the challenge and are now on the way to uncovering how the brain, aided by peripheral and central neurons, can sense and support the development and progression of cancer, a process that I could name “tumoriception.” Understanding cancer through the deciphering of the brain–tumor circuit will provide new insight into signaling mechanisms and thus pharmacological strategies that could block the pathological sprouting, remodeling, and hijacking of autonomic and sensory nerve fibers that lead to tumor-induced homeostatic disruption of the body.

And I think to myself what a wonderful world! (“*What a Wonderful World*” is a song written by Bob Thiele and George David Weiss. It was first recorded by Louis Armstrong and released in 1967 as a single.)
